# Exploring the value of routinely collected data on EQ-5D-5L and other electronic patient-reported outcome measures as prognostic factors in adults with advanced non-small cell lung cancer receiving immunotherapy

**DOI:** 10.1136/bmjonc-2023-000158

**Published:** 2024-05-15

**Authors:** Kuan Liao, David C Wong, Fabio Gomes, Corinne Faivre-Finn, Laura Moliner, Matthew Sperrin, Janelle Yorke, Sabine N van der Veer

**Affiliations:** 1 Centre for Health Informatics, Division of Informatics, Imaging and Data Sciences, Faculty of Biology, Medicine and Health, Manchester Academic Health Science Centre, The University of Manchester, Manchester, UK; 2 Department of Computer Science, The University of Manchester, Manchester, UK; 3 Medical Oncology Department, The Christie NHS Foundation Trust, Manchester, UK; 4 Division of Cancer Science, The University of Manchester, Manchester, UK; 5 The Christie NHS Foundation Trust, Manchester, UK; 6 Division of Nursing, Midwifery and Social Work, The University of Manchester, Manchester, UK; 7 Patient-Centred Research Centre, The Christie NHS Foundation Trust, Manchester, UK

**Keywords:** Immunotherapy, Lung cancer (non-small cell)

## Abstract

**Objective:**

Investigate whether routinely collected electronic patient-reported outcome measures (ePROMs) add prognostic value to clinical and tumour characteristics for adults with advanced non-small cell lung cancer (NSCLC) receiving immunotherapy.

**Methods and analysis:**

We retrospectively analysed data from adults with advanced NSCLC commencing immunotherapy between April 2019 and June 2022. Prognostic factors were ePROMs on quality of life (EuroQoL five-dimension five-level (EQ-5D-5L); EuroQoL Visual Analogue Scale (EQ-VAS)) and symptoms (patient-reported version of the Common Terminology Criteria for Adverse Events v5.0) completed at baseline and the first follow-up. We performed Cox proportional hazard regression for overall survival and time-to-progression as outcomes, and logistic regression for the onset of severe treatment toxicities (grade ≥3).

**Results:**

We included 379 patients; 161 (42.5%) completed ePROMs at baseline. Median overall survival and time-to-progression were 13.5 months (95% CI 11.3 to 16.7) and 10.5 months (95% CI 8.8 to 13.7), respectively. 36 (9.5%) experienced severe treatment toxicities during follow-up. Patients with lower EQ-5D-5L utility scores (HR per 0.1 unit increase 0.84, 95% CI 0.74 to 0.95) and higher symptom burden (HR 1.11; 95% CI 1.04 to 1.19) had poorer overall survival. This was also true for those with decreased EQ-VAS and increased symptom burden between baseline and the first follow-up. Lastly, only decreased EQ-5D-5L utility scores between baseline and the first follow-up were associated with shorter time-to-progression.

**Conclusion:**

ePROMs may add prognostic value to clinical and tumour characteristics for overall survival in adults with advanced NSCLC receiving immunotherapy.

**WHAT IS ALREADY KNOWN ON THIS TOPIC**

- Decision-making for adults with advanced non-small cell lung cancer (NSCLC) receiving immunotherapy requires prognostic information derived from both clinical and tumour characteristics and electronic patient-reported outcome measures (ePROMs).
- The ePROMs remain underused in the context of immunotherapy for advanced NSCLC.

**WHAT THIS STUDY ADDS**

- Baseline measurements of EuroQoL five-dimension five-level (EQ-5D-5L) utility score and symptom burden score derived from a patient-reported version of the Common Terminology Criteria for Adverse Events v5.0 were prognostic factors for overall survival.
- Changes in EuroQoL Visual Analogue Scale and symptom burden score at the first follow-up also have prognostic value for overall survival.
- Change in EQ-5D-5L utility score at the first follow-up is a prognostic factor for time-to-progression.

**HOW THIS STUDY MIGHT AFFECT RESEARCH, PRACTICE AND POLICY**

- ePROMs may provide prognostic information independently and in addition to clinical and tumour prognostic factors. Researchers should consider how to use ePROMs to predict survival outcomes accurately and further inform the decision-making in people with advanced NSCLC receiving immunotherapy.

## Introduction

 Lung cancer is the most common cause of cancer death in the UK in males and females.^1^ For many years, treatment options for people diagnosed with advanced non-small cell lung cancer (NSCLC) were limited to chemotherapy, which increased median survival time by 1.5 months compared with best supportive care.^2^ In the last decade, immunotherapy has revolutionised the treatment for advanced NSCLC^3^ with the availability of immune checkpoint inhibitors, such as atezolizumab (an anti-programmed death-ligand 1, anti-PD-L1 inhibitor) and pembrolizumab (an anti-programmed death 1, anti-PD-1 inhibitor). Randomised controlled trials showed that immunotherapy alone or in combination with chemotherapy compared with chemotherapy alone significantly improved clinical outcomes, such as response, survival and incidence of toxicities, in people with advanced NSCLC.^4^,^6^ However, guideline bodies have highlighted that immune checkpoint inhibitors for treatment of advanced NSCLC is an expensive treatment and that not all people respond to it.^7^ Furthermore, people with advanced NSCLC receiving immunotherapy may develop severe or even life-threatening treatment toxicities.^8^

In current clinical practice, guidelines for prescribing immunotherapy treatments are based on evidence from randomised trials.^9^ However, trials participants are often younger and healthier compared with patients that oncologists routinely see in their clinic. For example, the majority of clinical trials in people with advanced NSCLC receiving immunotherapy excluded those with an Eastern Cooperative Oncology Group (ECOG) performance status (PS) of 2 or higher,^10^ while this group makes up approximately 30% of patients with lung cancer in clinical practice. The difference of baseline clinical and tumour characteristics between trial cohorts and people attending oncology clinics may affect treatment response and tolerability.^11^,^13^ This means that clinical trial data only support decisions for prescribing immunotherapy in a selected group of people with advanced NSCLC.

To better understand who may benefit from immunotherapy and who may not, previous studies identified the predictive biomarker PD-L1 expression, and prognostic factors to support treatment decision-making, such as ECOG PS and neutrophil-to-lymphocyte ratio (NLR),^14^,^16^ or developed prognostic models incorporating clinical and blood biomarkers.^17^,^19^ However, patients with poor prognoses based on these factors or models may still benefit from immunotherapy.^15 20^ At the same time, severe treatment toxicities could result in discontinuation of immunotherapy and even death.^21 22^ This indicates a need for identifying further factors to provide prognostic information for guiding decisions on treating people with advanced NSCLC with immunotherapy.

Patient-reported outcome measures (PROMs) help to gain insight into symptom status, physical function, mental health and health-related quality of life (QoL) from the patient’s perspective.^23^ With the transition to more patient-centred care, routine collection of electronic PROMs (ePROMs) is now becoming more common in oncology practice.^24 25^ Although studies have reported PROM data in patients with advanced NSCLC receiving immune checkpoint inhibitors, most of these studies have focused on PROMs collected as part of a clinical trial.^26^,^28^ Only a few studies were conducted in routine care settings.^29^,^31^ Moreover, McLouth *et al*^29^ indicated that PROM values collected in clinical practice might differ from those in clinical trials.^29^ A systematic review by Efficace *et al*^32^ concluded that PROMs may have prognostic value for overall survival across cancer populations, including those with lung cancers.^32^ A subsequent scoping review of evidence on the prognostic value of PROMs in patients with NSCLC found that several studies reported the prognostic value of PROMs to predict other clinical outcomes.^33^ However, only a few of these used routinely collected PROM data sets, and even fewer investigated outcomes other than overall survival; none had been conducted using routinely collected PROMs in the context of immunotherapy.

Therefore, this study aimed to explore the prognostic value of routinely collected ePROMs in people with advanced NSCLC receiving immunotherapy, both at baseline and the first follow-up, for predicting survival and treatment toxicity to inform decisions among those patients on immunotherapy.

## Methods

We designed and conducted a prognostic factor study following the recommendations of the Prognosis Research Strategy (PROGRESS) 2: Prognostic Factor Research^34^ framework and reported this in accordance with REporting recommendations for tumour MARKer prognostic studies (REMARK) guideline.^35^

### Study context and data collection procedures

The context of this single-centre observational study was a large tertiary cancer hospital in England (UK). The hospital started integrating ePROM questionnaires into care pathways for patients with lung cancers in January 2019,^36^ implying that the ePROM data for the current analyses were collected as part of routine care rather than in the context of a research study. Patients were automatically enrolled in the ePROM service but could actively opt-out if they wished or could decide not to complete the ePROM survey at any time point. ePROM questionnaires included disease-specific symptom questions adapted using plain English from the Common Terminology Criteria for Adverse Events (CTCAE) version 5.0 and the EuroQoL five-dimension five-level questionnaire (EQ-5D-5L); we describe these instruments in more detail below. Enrolled patients received a text message with a web link to the ePROM questionnaires either at 17:00 on the day of a new patient appointment or 3 days before a scheduled follow-up appointment. They received immediate automated feedback on their responses on completion.^36^

### Study sample

The study retrospectively included consecutive people with advanced NSCLC who started immunotherapy between 19 April 2019 and 1 June 2022. People were included if they were (1) aged ≥18 years or older with advanced (or stage IV) NSCLC based on pathological confirmation; (2) commencing immunotherapy drugs (atezolizumab or pembrolizumab) alone or chemoimmunotherapy using atezolizumab or pembrolizumab as an immunotherapy regimen as any line of treatment. Eligible patients were all included in the study, regardless of whether they completed ePROMs or not.

### Outcomes of interest

Outcomes of interest included overall survival, time-to-progression and treatment toxicities. We defined *overall survival* as the length of time from initiating immunotherapy to the time of death from any cause or censored at the last day of follow-up. *Time-to-progression* was defined as the time from initiating immunotherapy to the time of documented disease progression and censored at the last clinical visit or the time of death from any cause. In the absence of response evaluation criteria in solid tumours data for most patients in our sample, we used a clinician-anchored approach to define the time of documented disease progression as the date of the first CT scan report mentioning progressive disease in the radiologist’s conclusion, or the date of the first clinical note stating progressive disease, when the CT scan report was not documented in the electronic patient record.^37^ Lastly, we defined *severe treatment toxicities* as the onset of any severe adverse events using the clinician-reported CTCAE v5.0.^38^ The CTCAE v5.0 lists relevant toxicities for people with lung cancer on systemic therapies as being absent or present; toxicities are graded based on their severity and frequency on a scale from 1 (mild) to 5 (death related to adverse events). We considered treatment toxicity severe if they had been graded 3 or higher.

### Demographic and clinical covariates at baseline

Online supplemental appendix 1 shows our selection of eight covariates reflecting baseline demographic and clinical characteristics. We selected these based on prognostic and prediction models from published studies^39 40^ and the expertise of the clinical members of our research team (CF-F, FG and JY). Data on covariates were extracted from the electronic patient record, considering a time window from 90 days before until 14 days after the start of immunotherapy treatment.

### ePROMs as prognostic factors of interest

As prognostic factors of interest, we considered four ePROMs measuring QoL and symptom burden, which we describe in more detail in online supplemental appendix 2. For ePROM scores at baseline, we considered data collected in the 6 weeks before starting immunotherapy treatment. For our early change in ePROM analyses, a change in ePROM was computed as the difference between the ePROM value at baseline and the corresponding value at the first follow-up visit during immunotherapy with the ePROM completed.

The *EQ-5D-5L* is a validated questionnaire to capture health-related QoL.^41 42^ It consists of five dimensions: mobility, self-care, usual activities, pain/discomfort and anxiety/depression, rating each dimension at five levels ranging from 1 (no/problems) to 5 (extreme/problems). To use the EQ-5D-5L as a prognostic factor, we calculated the utility score as per the standard value set for England, of which the values range from −0.285 (extreme/problems on all dimensions) to 1 (full health).^43^ The EuroQoL Visual Analogue Scale (*EQ-VAS*) asks people to indicate their overall health on a 0–100 hash-marked, vertical visual analogue scale, with 0 indicating the worst and 100 the best imaginable health.^41^

For symptom burden as a prognostic factor, we used an adapted, patient-reported version of the CTCAE v5.0 asking people in plain English to rate the presence and severity of 14 disease-specific symptoms including pain, swallowing, shortness of breath, cough, coughing up blood, tiredness, appetite loss, feeling sick, vomiting, diarrhoea, constipation, numbness, pins and needles or tingling in arms/legs/hands or feet, weakness in arms/legs/hands or feet and skin rash (see online supplemental appendix 3 for the full questionnaire). Severity grades were defined as: (1) it does not stop me from doing daily activities (coded as mild); (2) it stops me from doing daily activities (moderate); (3) as a result, I struggle to care for myself (severe). A grade of 0 was coded as ‘symptom absent’. The symptom items from the questionnaire were matched with items from the European Organisation for Research and Treatment of Cancer (EORTC) QoL questionnaire along with its lung cancer speciﬁc module (EORTC QLQ-C30/LC13) and validated in clinical practice.^44^ The questionnaire used to measure the presence and severity of the 14 disease-specific symptoms is presented in online supplemental appendix 3. We summed up the scores to compute the *symptom burden score* ranging between 0 and 42, with higher scores indicating higher symptom burden. As a sensitivity analysis, we used the *number of moderate to severe symptoms*, defined as the count of symptoms with a grade ≥2, reported by patients in the patient-reported version of the CTCAE v5.0. The value for this factor ranged from 0 to 14, with higher score indicating more moderate to severe symptoms.

### Missing data

Missing values in routinely collected data for the purpose of prognostic factor analysis can be handled in different ways, as missingness itself may provide information.^45^ Therefore, we performed three different methods to handle missing data for the analyses both at baseline and first follow-up: multiple imputation (in our main analyses), as well as complete case analysis and multiple imputation plus a ‘missing’ indicator for the prognostic factors of interest.

In the complete case analyses of baseline data, we only included patients with complete information at baseline; for the first follow-up analysis, we included those with complete information at baseline and first follow-up. Given the fraction of the missing values in the variables of interest was around 50%, we performed multivariate imputation by chained equations (MICE) using the information of both baseline characteristics and outcomes to iteratively impute 40 datasets.^46^,^48^ MICE assumes that the data are missing at random, which is a less restrictive assumption than complete case analysis, which requires missing completely at random. In addition, to account for potential informative missingness, we also explored the information of missing ePROMs at baseline and the first follow-up by incorporating a missing indicator with MICE.

### Descriptive analyses

We summarised baseline characteristics and both baseline and the first follow-up ePROMs as means with SDs for continuous variables with non-skewed distribution, as medians with IQR for skewed-distributed continuous variables, and as numbers with percentages for binary and categorical variables. We used Kaplan-Meier curves to describe the overall survival and time-to-progression. We compared characteristics and outcomes between baseline ePROM completers and non-completers to assess the risk of non-response bias.

### Modelling procedures

We performed Cox proportional hazard regression to predict overall survival and time-to-progression, and logistic regression to predict severe treatment toxicity using ePROM data at baseline and at the first follow-up visit during immunotherapy with completed ePROMs. We conducted separate analyses for each ePROM score in online supplemental appendix 2, so there were eight analyses in total (ie, four using the start of immunotherapy and four using the first follow-up as time zero). For each ePROM analysis, we fitted three models, adding up to a total of 24 models: (1) ePROM-only model, using only the ePROM score as a prognostic factor, (2) partially adjusted model, additionally adjusted for ECOG PS and PD-L1 score and (3) fully adjusted model, adjusted for the ePROM score and all nine prognostic factors (note that smoker vs non-smoker and ex-smoker vs non-smoker were considered as two separate prognostic factors) listed in online supplemental appendix 1.

To account for regression to the mean in the longitudinal multivariable cox regression analyses, we incorporated the baseline ePROM score, as well as an interaction term between change in ePROM score and months since baseline ePROM completion at the time of first ePROM completion during follow-up.

We conducted a sensitivity analysis in patients receiving first-line immunotherapy, as patients receiving first-line immunotherapy may have a different prognosis than those receiving non-first-line immunotherapy.^49^

We used R (V.4.2.0) for all analyses. All p values were two-sided, with a significance level of <0.05. All estimated statistics were reported with their 95% CIs.

### Sample size calculations

To estimate the minimum required sample size for our analyses, we used the ‘pmsampsize’ package in R, which follows the sample size criteria for developing multivariable prediction model recommended by Riley *et al*.^50^ Online supplemental appendix 4 shows the minimum required sample sizes for fully observed cases in all planned analyses. For the fully adjusted model of the survival outcomes, the minimum sample size required is 166 and 199 for the analyses at baseline and the first follow-up, respectively. The minimum sample size for the fully adjusted model of severe treatment toxicities is 1858 and 2229 for the analyses at baseline and the first follow-up, respectively. We considered findings for analyses hypothesis-generating if they had actual sample sizes smaller than those required.

### Patient and public involvement

Patients and members of public were not involved in this study.

## Results

### Description of baseline characteristics and ePROM scores, change in ePROM scores at the first follow-up and outcomes

#### Baseline characteristics and ePROMs

Table 1 summarises the baseline characteristics and ePROM scores of our study sample. We identified 379 patients treated between 19 April 2019 and 1 June 2022. The mean age was 66.3±10.3 years. The majority of patients (81.8%) had non-squamous cell carcinoma as their histological subtype, an ECOG PS of 0–1 (92.9%), and received immunotherapy as part of their first-line treatment (84.5%).

**Table 1:** Baseline demographics and ePROM values for the whole study sample, as well as compared between ePROM completers* and non-completers

Characteristic	Total (N=379)	Completers (n=161)	Non-completers (n=218)	P value†
*Demographics*				
Age (years), mean (SD)	66.3 (10.3)	64.6 (10.6)	67.5 (9.9)	0.006
Sex, n (%)				0.91
Female	179 (47.2)	75 (46.6)	104 (47.7)	
Male	200 (52.8)	86 (53.4)	114 (52.3)	
ECOG PS, n (%)‡				0.41
0	117 (30.9)	55 (34.2)	62 (28.4)	
1	235 (62.0)	93 (57.8)	142 (65.1)	
2	23 (6.0)	12 (7.5)	11 (5.0)	
3	4 (1.1)	1 (0.6)	3 (1.4)	
Smoking status, n (%)				0.07
Current smoker	96 (25.3)	32 (19.9)	64 (29.4)	
Ex-smoker	252 (66.5)	113 (70.2)	139 (63.8)	
Life-long never	30 (7.9)	16 (9.9)	14 (6.4)	
Missing	1 (0.3)	0	1 (0.5)	
PD-L1 score group, n (%)				0.14
<1%	118 (31.1)	57 (35.4)	61 (28.0)	
≥1%, <50%	79 (20.8)	35 (21.7)	44 (20.2)	
≥50%	158 (41.7)	58 (36.0)	100 (45.9)	
Missing	24 (6.3)	11 (6.8)	13 (6.0)	
NLR, mean (SD)	6.9 (5.5)	6.9 (5.8)	6.9 (5.2)	0.98
Missing NLR, n (%)	22 (5.8)	2 (1.2)	20 (9.2)	0.002
Histology				0.83
Non-squamous cell carcinoma	310 (81.8)	133 (82.6)	177 (81.2)	
Squamous cell carcinoma	69 (18.2)	28 (17.4)	41 (18.8)	
Line of immunotherapy				0.32
First-line	320 (84.4)	132 (82.0)	188 (86.2)	
Not first-line	59 (15.6)	29 (18.0)	30 (13.8)	
Treatment				0.16
Immunotherapy	196 (51.7)	76 (47.2)	120 (55.0)	
Immuno-chemotherapy	183 (48.3)	85 (52.8)	98 (45.0)	
*ePROM scores* §				
EQ-5D-5L utility score, median (IQR)		0.81 (0.72–0.92)		
EQ-VAS score, median (IQR)		70 (50–80)		
Symptom burden score, median (IQR)		3 (2–5)		

* ePROMs completers were people who completed ePROMs within 6 weeks before starting immunotherapy.

† Hypothesis testing for the difference between ePROMs completers and non-completers. χ2 tests for sex, smoking history, PD-L1 score group, missing NLR, histology and line of immunotherapy. Two sample t-test for age and NLR. Wilcoxon rank sum test for ECOG PS.

‡ The distribution of ECOG PS scores in our study population was similar to that of other studies.^60 61^

§ No comparison available because ePROM non-completers did not have ePROM scores at baseline.

ECOG PS, Eastern Cooperative Oncology Group performance status; ePROM, electronic patient-reported outcome measure; EQ-5D-5L, EuroQoL five-dimension five-level; EQ-VAS, EuroQoL Visual Analogue Scale; NLR, neutrophil-to-lymphocyte ratio; PD-L1, programmed death-ligand 1.

Of the 379 patients, 161 (42.5%) patients completed the ePROM questionnaire before the start of the immunotherapy. These completers were significantly younger and had a significantly lower proportion of missing NLR, compared with the non-completers (table 1).

##### Change in ePROMs scores at the first follow-up

Among the 161 completers of a baseline ePROM, 134 patients completed a follow-up ePROM at least once during immunotherapy. Table 2 shows that the median time between baseline and first ePROM response during immunotherapy was 0.82 months (IQR 0.67–1.37 months) and that apart from the EQ-5D-5L utility score, ePROM scores changed significantly between baseline and follow-up, all reflecting a deterioration of patients’ health status.

**Table 2:** Absolute change of ePROMs between baseline and the first follow-up* (n=134)

ePROM	Mean difference in score (95% CI)	P value†
EQ-5D utility score	−0.02 (-0.05 to 0.01)	0.26
EQ-VAS	−5.81 (−10.54 to 1.09)	0.02
Symptom burden score	1.31 (0.70 to 1.93)	<0.001

* Median months between baseline and the first ePROM response during follow-up was 0.82 (IQR 0.67–1.37).

† T-test of the mean difference in score between baseline and the first follow-up.

ePROM, electronic patient-reported outcome measure; EQ-5D, EuroQoL five dimension; EQ-VAS, EuroQoL Visual Analogue Scale.

### Outcomes

**Figure 1:**
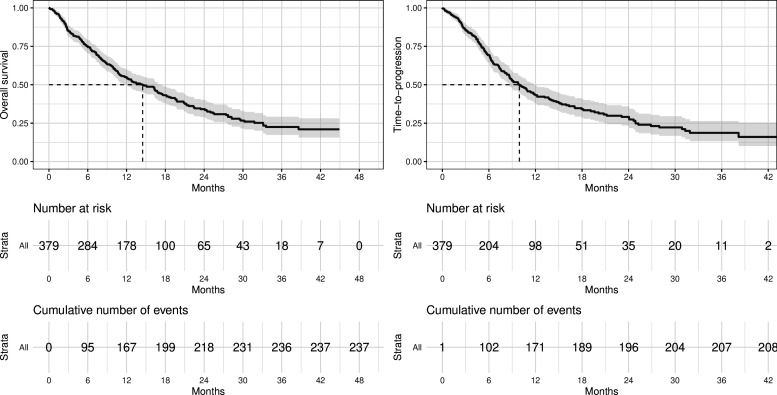
Overall survival (left panel) and time-to-progression (right panel) in people with advanced non-small cell lung cancer receiving immunotherapy.

With the median follow-up (censoring) time of 24.5 months (95% CI 19.7 to 30.2) and 142 (37.5%) patients censored, 237 of 379 patients (62.5%) had died, 208 (54.9%) had disease progression, and 36 (9.5%) had experienced severe treatment toxicities. The median overall survival was 13.5 months (95% CI 11.3 to 16.7) and the median time-to-progression was 10.5 months (95% CI 8.8 to 13.7) (figure 1).

There was no difference between ePROM completers and non-completers for overall survival but that may be not true for time-to-progression, although the difference was not statistically significant. Online supplemental appendix 5 shows that completers had a median overall survival of 13.5 months (95% CI 10.3 to 19.8 months) versus 14.1 months (95% CI 10.3 to 19.8 months) in non-completers (p=0.89), while the completers had a shorter median time-to-progression of 8.7 months (95% CI 6.5 to 12.2 months) versus 11.9 months for non-completers (95% CI 9.8 to 19.6 months, p=0.21). There was also no difference between completers and non-completers for severe treatment toxicities (9.9% vs 9.2%, χ^2^=0.005, p=0.94).

### Prognostic value of baseline ePROMs scores

Table 3 presents the pooled HRs or ORs obtained from fully adjusted models using multiple imputation for both baseline ePROMs and change in ePROMs to predict overall survival, time-to-progression and severe treatment toxicities. All ePROMs were associated with overall survival in the fully adjusted ePROM analyses except for EQ-VAS with a borderline insignificant association (HR per 10 unit increase 0.92; 95% CI 0.85 to 1.00). Patients with a lower EQ-5D-5L utility score (HR per 0.1 unit increase 0.84, 95% CI 0.75 to 0.94) or higher symptom burden score (HR 1.13; 95% CI 1.04 to 1.22) had poorer overall survival. Forest plots of the fully adjusted models for predicting overall survival, including the HRs for the baseline ePROM and the clinical parameters, are shown in online supplemental appendix 6. The complete case analyses and those using multiple imputation with a missing indicator produced similar findings (online supplemental appendix 7). None of the baseline ePROMs were significantly associated with time-to-progression and severe treatment toxicities, regardless of how we addressed the missing data (online supplemental appendices 8 and 9).

**Table 3:** Prognostic value of baseline ePROMs and change in ePROM between baseline and first completion at the first follow-up in the fully adjusted models for overall and time-to-progression

Prognostic factor of interest	Overall survival		Time-to-progression	
	HR (95% CI)	P value	HR (95% CI)	P value
*Baseline ePROM*				
EQ-5D-5L utility score (per 0.1 unit increase)	0.84 (0.74 to 0.95)	0.009	1.06 (0.91 to 1.23)	0.48
EQ-VAS (per 10 unit increase)	0.92 (0.85 to 1.00)	0.06	0.98 (0.90 to 1.06)	0.59
Symptom burden score	1.13 (1.04 to 1.22)	0.004	0.92 (0.77 to 1.10)	0.37
*Change in ePROM*				
EQ-5D-5L utility score (per 0.1 unit increase)	0.89 (0.76 to 1.05)	0.17	0.84 (0.73 to 0.97)	0.02
EQ-VAS (per 10 unit increase)	0.89 (0.81 to 0.97)	0.01	0.96 (0.88 to 1.06)	0.43
Symptom burden score	1.09 (1.03 to 1.15)	0.008	1.07 (1.00 to 1.14)	0.04

HRs were obtained from the pooled estimates after multiple imputation.

The REporting recommendations for tumour MARKer prognostic studies checklist is presented in online supplemental appendix 13.

ePROM, patient-reported outcome measure; EQ-5D-5L, EuroQoL five-dimension five-level; EQ-VAS, EuroQoL Visual Analogue Scale.

### Prognostic value of early change in ePROM scores

In table 3, a decrease in EQ-VAS (HR per 10 unit increase 0.89; 95% CI 0.81 to 0.97), an increase in symptom burden score (HR 1.09; 95% CI 1.03 to 1.15) and an increase in number of moderate to severe symptoms (HR 1.16; 95% CI 1.01 to 1.35) were associated with poorer overall survival in the fully adjusted analyses, regardless of how we handled missing data (online supplemental appendix 7). Forest plots of the fully adjusted models for change in ePROMs for predicting overall survival are presented in online supplemental appendix 10.

An increase in EQ-5D utility score (HR per 0.1 unit increase, 0.84; 95% CI 0.73 to 0.97) was associated with longer time-to-progression in the fully adjusted analysis (table 3). This association became borderline statistically significant in the fully adjusted analysis for a decrease in the symptom burden score (HR, 1.07; 95% CI 1.00 to 1.14), with similar but borderline insignificant result in the (sufficiently powered) partially adjusted analysis (HR 1.05; 95% CI 0.996 to 1.11) (online supplemental appendix 8). Findings were similar for the complete case analyses and those using multiple imputation with a missing indicator (online supplemental appendix 8). Forest plots of the fully adjusted models of change in ePROMs for predicting time-to-progression are presented in online supplemental appendix 11. None of the change in ePROM scores was associated with the severe treatment toxicities, no matter how the missing data were handled (online supplemental appendix 9).

Similar results were found if we summarised the symptom burden ePROM as the number of moderate to severe symptoms (online supplemental appendices 7–9). The sensitivity analysis where we included only patients who received first-line immunotherapy (n=320, table 1) showed largely similar results (online supplemental appendix 12).

## Discussion

This study provided insights into the potential prognostic value of routinely collected ePROMs in addition to clinical and tumour characteristics in adults with advanced NSCLC receiving immunotherapy. We found that the baseline measurements of EQ-5D-5L utility score and symptom burden ePROMs were prognostic factors for overall survival, with change in EQ-VAS and symptom burden score at the first follow-up also having prognostic value for this outcome. Apart from the change in EQ-5D-5L utility score being a prognostic factor for time-to-progression, none of the other baseline or change in ePROM scores showed a significant association with time-to-progression or severe treatment toxicities.

Previous reviews demonstrated the potential of PROMs as prognostic factors for overall survival for people with NSCLC receiving radiotherapy, chemotherapy or targeted therapy.^32 33 51^ In addition, Hopkins *et al*^52^ concluded that ePROMs may have prognostic value for predicting overall survival and time-to-progression in patients receiving immunotherapy in a clinical trial setting and could therefore serve as a prognostic factor for stratifying trial participants.^52^ Our study confirmed these findings in routine practice settings for people with NSCLC receiving immunotherapy, thereby adding insights into the value of ePROMs to guide real-world treatment decisions in the current era of new systemic anticancer therapy. Future model development studies should therefore consider including ePROMs in multivariable clinical prediction models for overall survival in this population and evaluate whether ePROMs enhance the models’ predictive performance.

Different to most other studies included in the 2022 scoping review by Liao *et al*,^33^ we also explored the prognostic value of ePROMs for predicting onset of severe treatment toxicities in this population but found no associations. In contrast, Iivanainen *et al*^53^ developed a machine-learning model that did suggest ePROMs may be associated with this outcome in people with NSCLC treated with immunotherapy.^53^ However, they did not adjust for clinical and tumour characteristics and did not account for the fact that severe treatment toxicities are a rare (and therefore imbalanced) outcome, making the predictive performance measures of their model potentially misleading.^54^ Yet, ePROMs may help to identify presence of treatment toxicities by facilitating timely capturing of worsening side effects in people while on immunotherapy.^55^ The potential diagnostic value of ePROMs for severe treatment toxicities may therefore warrant further studies.

In contrast to a previous study reporting an improvement in symptom burden and QoL after 6 weeks of starting immunotherapy and other palliative lung cancer therapies,^56^ we found a deterioration in these patient-reported aspects after 3–4 weeks. Future analyses may therefore explore if and how timing of ePROM follow-up influences the prognostic value of a longitudinal change in ePROM scores.

Our study has several limitations. First, our sample size for the fully adjusted analyses for overall survival and time-to-progression did not meet the requirements, and the findings from these should thus be considered as hypothesis generating. Yet, for overall survival and time-to-progression, we had sufficient statistical power in the partially adjusted analyses, where we observed similar results. Moreover, our sample size was close to the minimum sample size criteria, especially in the baseline analyses. Although we had initial interest in severe treatment toxicities, the small sample size and number of events did not allow us to draw any robust conclusion or generate any hypotheses for that outcome. Alternative outcomes such as time to next treatment and time to treatment discontinuation could be considered in future research.^57 58^ The limited sample size did not allow us to explore the prognostic value of individual patient-reported symptoms listed in our symptom burden ePROM or adjust for more potential prognostic factors in the analysis, such as comorbidity or whether patients had advanced NSCLC as a primary diagnosis (vs a relapse or secondary cancer). Second, we defined time-to-progression based on imaging results available in the hospital’s electronic health record system. Consequently, patients discharged to other healthcare providers and for whom we did not have access to all imaging data, were right censored at the date of the last clinical visit. Although we assumed the right censoring was independent of disease progression, we acknowledge that the right censoring could also have been informative. In addition, for the overall survival outcome, a proportion of patients who were lost to follow-up or alive at the end of the data collection were also censored. This means we may have underestimated the number of disease progression cases and reduced statistical power, but this is unlikely to have affected our overall findings because the Cox proportional hazard regression accounts for the right censoring by the maximum partial likelihood estimation, which compares hazard rates while considering censored observations. Third, a large proportion of patients in our sample had missing ePROM data, which reduced the accuracy of our estimates of the prognostic value of ePROMs. However, since our findings were similar regardless of how we handled missing data, this provided some reassurance that having a more complete data set would not change our overall conclusions. Fourth, this study retrospectively analysed routinely collected data from a single centre in England, UK, limiting the generalisability of our findings. Future research should, therefore, externally validate our results by replicating our analyses in prospective populations treated in different (types of) centres and healthcare systems. Lastly, we used an adapted ePROM to measure symptom burden that is not widely used in other cancer centres, limiting the generalisability of our findings to contexts that use different symptom ePROMs.^59^

## Conclusion

This study demonstrated that routinely collected ePROMs (based on EQ-5D-5L and symptom burden) before therapy and changes in ePROMs on therapy (based on EQ-VAS and symptom burden) have added prognostic value in adults with advanced NSCLC receiving immunotherapy. This warrants future development and evaluation of clinical prediction models incorporating routinely collected ePROMs as prognostic factors to help identify people with advanced NSCLC who are likely to have good overall survival with immunotherapy. Future studies with larger sample sizes should assess the prognostic value of routinely collected ePROMs for predicting time-to-progression and severe treatment toxicities in this population.

## Supplementary material

10.1136/bmjonc-2023-000158 online supplemental file 1

## Data Availability

No data are available.

## References

[1] Cancer Research UK (2018). Lung cancer Statistics. https://www.cancerresearchuk.org/health-professional/cancer-statistics/statistics-by-cancer-type/lung-cancer.

[2] NSCLC Meta-Analyses Collaborative Group (2008). Chemotherapy in addition to supportive care improves survival in advanced non–small-cell lung cancer: A systematic review and meta-analysis of individual patient data from 16 randomized controlled trials. JCO.

[3] Arbour KC, Riely GJ (2019). Systemic therapy for locally advanced and metastatic non–small cell lung cancer: a review. JAMA.

[4] Rittmeyer A, Barlesi F, Waterkamp D (2017). Atezolizumab versus Docetaxel in patients with previously treated non-small-cell lung cancer (OAK): a phase 3, open-label, Multicentre randomised controlled trial. The Lancet.

[5] Reck M, Rodríguez-Abreu D, Robinson AG (2016). Pembrolizumab versus chemotherapy for PD-L1–positive non–small-cell lung cancer. N Engl J Med.

[6] Borghaei H, Langer CJ, Paz-Ares L (2020). Pembrolizumab plus chemotherapy versus chemotherapy alone in patients with advanced non–small cell lung cancer without tumor PD-L1 expression: A pooled analysis of 3 randomized controlled trials. Cancer.

[7] NICE (2023). NICE guidance. https://www.nice.org.uk/guidance/conditions-and-diseases/cancer/lung-cancer/products?ProductType=Guidance&Status=Published.

[8] Li Y, Zhang Y, Jia X (2021). Effect of immune-related adverse events and Pneumonitis on prognosis in advanced non–small cell lung cancer: A comprehensive systematic review and meta-analysis. Clin Lung Cancer.

[9] Planchard D, Popat S, Kerr K (2018). Metastatic non-small cell lung cancer: ESMO clinical practice guidelines for diagnosis, treatment and follow-up. Ann Oncol.

[10] Rashdan S, Gerber DE (2019). Immunotherapy for non-small cell lung cancer: from clinical trials to real-world practice. Transl Lung Cancer Res.

[11] Lilenbaum RC, Cashy J, Hensing TA (2008). Prevalence of poor performance status in lung cancer patients: implications for research. J Thorac Oncol.

[12] Altundag O, Stewart DJ, Fossella FV (2007). Many patients 80 years and older with advanced non-small cell lung cancer (NSCLC) can tolerate chemotherapy. J Thorac Oncol.

[13] Tournoy KG, Thomeer M, Germonpré P (2018). Does Nivolumab for progressed metastatic lung cancer fulfill its promises? an efficacy and safety analysis in 20 general hospitals. Lung Cancer.

[14] Brody R, Zhang Y, Ballas M (2017). PD-L1 expression in advanced NSCLC: insights into risk stratification and treatment selection from a systematic literature review. Lung Cancer.

[15] Dall’Olio FG, Maggio I, Massucci M (2020). ECOG performance status ≥2 as a Prognostic factor in patients with advanced non small cell lung cancer treated with immune Checkpoint inhibitors—A systematic review and meta-analysis of real world data. Lung Cancer.

[16] Brueckl WM, Ficker JH, Zeitler G (2020). Clinically relevant Prognostic and predictive markers for immune-Checkpoint-inhibitor (ICI) therapy in non-small cell lung cancer (NSCLC). BMC Cancer.

[17] Mezquita L, Auclin E, Ferrara R (2018). Association of the lung immune Prognostic index with immune Checkpoint inhibitor outcomes in patients with advanced non–small cell lung cancer. JAMA Oncol.

[18] Moor R, O’Byrne K, Roberts K (2019). Modified lung immune predictive index (mLIPI) as a predictive tool of Nivolumab outcomes and immune related adverse events in advanced non-small cell lung cancer (NSCLC) patients. Lung Cancer.

[19] Prelaj A, Rebuzzi SE, Pizzutilo P (2020). Epsilon: A Prognostic score using clinical and blood biomarkers in advanced non–small-cell lung cancer treated with Immunotherapy. Clin Lung Cancer.

[20] Doroshow DB, Bhalla S, Beasley MB (2021). PD-L1 as a biomarker of response to immune-Checkpoint inhibitors. Nat Rev Clin Oncol.

[21] Durrechou Q, Domblides C, Sionneau B (2020). Management of immune Checkpoint inhibitor toxicities. Cancer Manag Res.

[22] Puzanov I, Diab A, Abdallah K (2017). Managing toxicities associated with immune Checkpoint inhibitors: consensus recommendations from the society for Immunotherapy of cancer (SITC). *J Immunother Cancer*.

[23] Nelson EC, Eftimovska E, Lind C (2015). Patient reported outcome measures in practice. BMJ.

[24] Bouazza YB, Chiairi I, El Kharbouchi O (2017). Patient-reported outcome measures (Proms) in the management of lung cancer: A systematic review. Lung Cancer.

[25] Tjong MC, Doherty M, Tan H (2021). Province-wide analysis of patient-reported outcomes for stage IV non-small cell lung cancer. Oncologist.

[26] Barlesi F, Garon EB, Kim D-W (2019). Health-related quality of life in KEYNOTE-010: a phase II/III study of Pembrolizumab versus Docetaxel in patients with previously treated advanced, programmed death ligand 1–expressing NSCLC. J Thorac Oncol.

[27] Brahmer JR, Rodríguez-Abreu D, Robinson AG (2017). Health-related quality-of-life results for Pembrolizumab versus chemotherapy in advanced, PD-L1-positive NSCLC (KEYNOTE-024): a Multicentre, International, randomised, open-label phase 3 trial. The Lancet Oncology.

[28] Reck M, Taylor F, Penrod JR (2018). Impact of Nivolumab versus Docetaxel on health-related quality of life and symptoms in patients with advanced squamous non–small cell lung cancer: results from the Checkmate 017 study. Journal of Thoracic Oncology.

[29] Steffen McLouth LE, Lycan TW, Jr BJ (2020). Patient-reported outcomes from patients receiving Immunotherapy or Chemo-Immunotherapy for metastatic non-small cell lung cancer in clinical practice. Clinical Lung Cancer.

[30] Presley CJ, Arrato NA, Janse S (2021). Functional disability among older versus younger adults with advanced non-small-cell lung cancer. *JCO Oncol Pract*.

[31] Valentine TR, Presley CJ, Carbone DP (2022). Illness perceptions and psychological and physical symptoms in newly diagnosed lung cancer. *Health Psychology*.

[32] Efficace F, Collins GS, Cottone F (2021). Patient-reported outcomes as independent Prognostic factors for survival in oncology: systematic review and meta-analysis. Value Health.

[33] Liao K, Wang T, Coomber-Moore J (2022). Prognostic value of patient-reported outcome measures (Proms) in adults with non-small cell lung cancer: a Scoping review. BMC Cancer.

[34] Riley RD, Hayden JA, Steyerberg EW (2013). Prognosis research strategy (PROGRESS) 2: Prognostic factor research. PLOS Med.

[35] McShane LM, Altman DG, Sauerbrei W (2005). Reporting recommendations for tumour marker Prognostic studies (REMARK). Br J Cancer.

[36] Crockett C, Gomes F, Faivre-Finn C (2021). The routine clinical implementation of electronic patient-reported outcome measures (ePROMs) at the Christie NHS foundation trust. Clinical Oncology.

[37] Griffith SD, Tucker M, Bowser B (2019). Generating real-world tumor burden endpoints from electronic health record data: comparison of RECIST, Radiology-anchored, and clinician-anchored approaches for abstracting real-world progression in non-small cell lung cancer. Adv Ther.

[38] National Cancer Institute (2017). Common terminology criteria for adverse events (CTCAE) | protocol development | CTEP. https://ctep.cancer.gov/protocoldevelopment/electronic_applications/ctc.htm.

[39] Prelaj A, Boeri M, Robuschi A (2022). Machine learning using real-world and Translational data to improve treatment selection for NSCLC patients treated with Immunotherapy. Cancers (Basel).

[40] Sakata Y, Kawamura K, Ichikado K (2019). The association between tumor burden and severe immune-related adverse events in non-small cell lung cancer patients responding to immune-Checkpoint inhibitor treatment. Lung Cancer.

[41] Herdman M, Gudex C, Lloyd A (2011). Development and preliminary testing of the new five-level version of EQ-5D (EQ-5D-5L). *Qual Life Res*.

[42] Feng Y, Devlin N, Herdman M (2015). Assessing the health of the general population in England: how do the Three- and five-level versions of EQ-5D compare. Health Qual Life Outcomes.

[43] Devlin NJ, Shah KK, Feng Y (2018). Valuing health-related quality of life: an EQ-5D-5L value set for England. Health Econ.

[44] Christodoulou M, McCloskey P, Stones N (2014). Investigation of a patient reported outcome tool to assess radiotherapy-related toxicity prospectively in patients with lung cancer. Radiother Oncol.

[45] Sperrin M, Martin GP, Sisk R (2020). Missing data should be handled differently for prediction than for description or causal explanation. J Clin Epidemiol.

[46] Marshall A, Altman DG, Holder RL (2010). Comparison of imputation methods for handling missing Covariate data when fitting a Cox proportional hazards model: a Resampling study. BMC Med Res Methodol.

[47] White IR, Royston P, Wood AM (2011). Multiple imputation using chained equations: issues and guidance for practice. Stat Med.

[48] Graham JW, Olchowski AE, Gilreath TD (2007). How many Imputations are really needed? some practical Clarifications of multiple imputation theory. Prev Sci.

[49] Wu S, Wang L, Li W (2021). Comparison between the first-line and second-line Immunotherapy drugs in the progression-free survival and overall survival in advanced non-small cell lung cancer: a systematic review and meta-analysis of randomized controlled trials. Ann Palliat Med.

[50] Riley RD, Snell KI, Ensor J (2019). Minimum sample size for developing a multivariable prediction model: PART II - binary and time-to-event outcomes. Stat Med.

[51] Mierzynska J, Piccinin C, Pe M (2019). Prognostic value of patient-reported outcomes from international randomised clinical trials on cancer: a systematic review. Lancet Oncol.

[52] Hopkins AM, Wagner J, Kichenadasse G (2020). Patient-reported outcomes as a Prognostic marker of survival in patients with advanced Nonsmall cell lung cancer treated with Immunotherapy. Int J Cancer.

[53] Iivanainen S, Ekstrom J, Virtanen H (2021). Electronic patient-reported outcomes and machine learning in predicting immune-related adverse events of immune Checkpoint inhibitor therapies. BMC Med Inform Decis Mak.

[54] Chicco D, Jurman G (2020). The advantages of the Matthews correlation coefficient (MCC) over F1 score and accuracy in binary classification evaluation. BMC Genomics.

[55] Lai-Kwon J, Cohen JE, Lisy K (2023). The feasibility, acceptability, and effectiveness of electronic patient-reported outcome symptom monitoring for immune Checkpoint inhibitor toxicities: A systematic review. *JCO Clin Cancer Inform*.

[56] Tournoy KG, Adam V, Muylle I (2023). Health outcomes with curative and palliative therapies in real world: role of the quality of life summary score in Thoracic oncology patients. Cancers (Basel).

[57] Walker B, Boyd M, Aguilar K (2021). Comparisons of real-world time-to-event end points in oncology research. *JCO Clin Cancer Inform*.

[58] Griffith SD, Miksad RA, Calkins G (2019). Characterizing the feasibility and performance of real-world tumor progression end points and their association with overall survival in a large advanced non–small-cell lung cancer data set. *JCO Clinical Cancer Informatics*.

[59] Spencer KL, Absolom KL, Allsop MJ (2023). Fixing the leaky pipe: how to improve the uptake of patient reported outcomes-based Prognostic and predictive models in cancer clinical practice. JCO Clin Cancer Inform.

[60] Cramer-van der Welle CM, Verschueren MV, Tonn M (2021). Real-world outcomes versus clinical trial results of Immunotherapy in stage IV non-small cell lung cancer (NSCLC) in the Netherlands. Sci Rep.

[61] Mezquita L, Preeshagul I, Auclin E (2021). Predicting Immunotherapy outcomes under therapy in patients with advanced NSCLC using dNLR and its early Dynamics. Eur J Cancer.

