# Supplementary material for: Exploring the value of routinely collected data on EQ-5D-5L and other electronic patient-reported outcome measures as prognostic factors in adults with advanced non-small cell lung cancer receiving immunotherapy
Source: BMJ Oncol. 2024 May 15;3(1):e000158. doi: 10.1136/bmjonc-2023-000158 (PMC11203075; doi:10.1136/bmjonc-2023-000158)
Supplement: online supplemental file 1 [file bmjonc-2023-000158supp001.pdf]

Appendices

Appendix 1. Selected demographic and clinical covariates at baseline

Covariate class	Covariate variable	Variable type	Description
Clinical characteristics	Age	Continuous	Age at the start of immunotherapy.
	Sex	Categorical	Patient's sex: male, female.
	ECOG performance status	Discrete (treated as continuous in the analyses)	Performance status rated by the clinical team. Six-point scale ranging from 0 (fully active) to 5 (dead), with 4 indicating completely disabled.
	Smoking status*	Categorical	Patient's smoking status: smoker, ex-smoker, non-smoker.
Laboratory tests	Neutrophil-to-lymphocyte ratio (NLR)	Continuous	Calculated by dividing the number of neutrophils by the number of lymphocytes in the blood sample.
Tumour characteristics	PD-L1 tumour proportion score	Continuous	PD-L1 tumour proportion score reported as a percentage on a scale of 0% to 100%
	Histology	Categorical	Histology type of the tumour: Squamous, non-squamous.
Treatment	Line of immunotherapy	Categorical	Treatment line of the immunotherapy: first-line, any other lines.

Abbreviations: ECOG - Eastern Cooperative Oncology Group; NLR - Neutrophil-to-lymphocyte ratio; PD-L1 - programmed death ligand 1.
* In sample size calculation for regression analyses, smoking status was considered by including two variables in the model: smoker vs. non-smoker and ex-smoker vs. non-smoker.

Appendix 2. An overview of ePROMs as the prognostic factors of interest

ePROM	Prognostic factor	Definition and theoretical range
EQ-5D-5L	EQ-5D-5L utility score	An individual's overall health status and the impact of health conditions on their daily functioning.<br>Range: -0.285 (extreme/problems on all dimensions) to 1 (full health).
	Change in EQ-5D-5L utility score*	Change in EQ-5D-5L utility score from baseline to first ePROM completion during follow-up.<br>Range: -1.285 to 1.285. An increase in EQ-5D-5L utility score indicated an improvement in health-related quality of life.
EQ-VAS	EQ-VAS	A component of the EQ-5D questionnaire that measures an individual's self-rated health on a visual analog scale.<br>Range: 0 (worst imaginable health state) to 100 (best imaginable health state).
	Change in EQ-VAS*	Change in EQ-VAS from baseline to first ePROM completion during follow-up.<br>Range: -100 to 100. An increase in EQ-VAS indicated an improvement in health-related quality of life.
Patient-reported version of the CTCAE v5.0	Symptom burden score	To what extent a person’s life is impacted by 14 lung cancer-specific symptoms.<br>Range: 0 (no symptoms) to 42 (all 14 symptoms were severe).
	Change in overall symptom burden score*	Change in overall symptom burden score from baseline to first ePROM completion during follow-up.<br>Range: -84 to 84. An increase in overall symptom burden score indicated a greater impact on a person's life due to the presence and severity of symptoms.
	Number of moderate to severe symptoms†	Number of moderate or severe lung cancer-specific symptoms.<br>Range: 0 (no moderate to severe symptoms) to 14 (all 14 disease-specific symptoms were moderate to severe).
	Change in number of moderate to severe symptoms*†	Change in number of moderate to severe symptoms from baseline to first ePROM completion during follow-up.<br>Range: -28 to 28. An increase in number of moderate to severe symptoms indicated the severity of lung cancer-specific symptoms had worsened for an individual.

Abbreviations: ePROM – electronic Patient-reported outcome measures; EQ-5D-5L – EuroQoL five dimension five level; EQ-VAS – EuroQoL Visual Analogue Scale; CTCAE – Common Terminology Criteria for Adverse Events.

* In the analyses of the first follow-up, we adjusted for the corresponding baseline ePROM score and an interaction term between change in ePROM score and months since baseline ePROM completion at the time of first ePROM completion during follow-up.

†The number of moderate to severe symptoms is supplementary to the symptom burden score of summarising the Patient-reported version of the CTCAE v5.0

Appendix 3. Severity levels of the 14 lung-cancer specific symptoms adapted from the Common Terminology Criteria for Adverse Events (CTCAE).

	No symptom (coded as 0)	Mild (coded as 1)	Moderate (coded as 2)	Severe (coded as 3)
Pain in chest, throat, neck back or abdomen	I do not have any pain in any of the areas	I am still able to do my routine activities (for example light housework, deskwork or shopping)	It limits me doing my routine activities (for example light housework, deskwork or shopping)	It limits me caring for myself (for example washing or dressing)
Difficulty swallowing	No	I am still able to eat my normal diet	I have had to change my diet to easier-to-eat foods	I have to use a feeding tube and/or had to be admitted to hospital since the last review appointment for my cancer
Shortness of breath	No	I become short of breath with moderate effort	I become short of breath with minimal effort; this limits me from doing my routine activities (for example light housework, deskwork or shopping)	I am short of breath at rest; this limits me caring for myself (for example washing or dressing)
Cough	No	I have not required medical help	It limits me doing my routine activities (for example light housework, deskwork or shopping) and/or have required medical help	It limits me caring for myself (for example washing or dressing)
Cough up blood	No	I have not required medical help	I have required medical help	I had to be admitted to hospital since the last review appointment for my cancer

Tiredness	No	It is relieved by rest	It is not relieved by rest and / or it limits me doing my routine activities (for example light housework, deskwork or shopping)	It is not relieved by rest and / or it limits me caring for myself (for example washing or dressing)
Appetite loss	No	I still eat as normal	I eat less than normal and/or I have been prescribed food supplements	I eat very little and/or I had to be admitted to hospital since the last review appointment for my cancer
Feeling sick	No	My appetite has reduced but I am still eating normally	I am eating less but have not lost any weight	I am eating and drinking very little and/or had to be admitted to hospital since the last review appointment for my cancer
Vomiting	No	1-2 times in a 24-hour period	3-5 times in a 24-hour period	6 or more times and/or I had to be admitted to hospital
Diarrhoea	No	1-3 times more than normal	4-6 times more than normal and/or it limits me doing my routine activities (for example light housework, deskwork or shopping)	7 times or more than usual and or because of this I was admitted to hospital since the last review appointment for my cancer and/or it limits me caring for myself (for example washing or dressing)

Constipation	No	I take occasional laxatives but I'm still able to do my routine activities (for example light housework, deskwork or shopping)	I take regular laxatives and/or it limits me doing my routine activities (for example light housework, deskwork or shopping)	It limits me caring for myself (for example washing or dressing)
Numbness, pins and needles or tingling in arms/legs/hands or feet	No	I am still able to do my routine activities (for example light housework, deskwork or shopping)	It limits me doing my routine activities (for example light housework, deskwork or shopping)	It limits me caring for myself (for example washing or dressing)
Weakness in your arms/legs/hands or feet	No	I am still able to do my routine activities (for example light housework, deskwork or shopping)	It limits me doing my routine activities (for example light housework, deskwork or shopping)	It limits me caring for myself (for example washing or dressing)
Skin rash	No	It covers less than a tenth of my body and may feel itchy, burning or tight. I am still able to do my routine activities (for example light housework, deskwork or shopping)	It covers between a tenth and a third of my body and/or it limits me doing my routine activities (for example light housework, deskwork or shopping)	It covers more than a third of my body. It limits me caring for myself (for example washing or dressing) and/or because of this I have required a procedure

Appendix 4. Minimum required sample sizes for fully observed cases in the different types of analyses.

Type of model	**ePROM-only**		**Partially adjusted**		**Fully adjusted**	
Type of analysis / ***Outcome***	*Baseline* (n=1) *	*First follow-up* (n=3) *	*Baseline* (n=3) *	*First follow-up* (n=5) *	*Baseline* (n=10) *	*First follow-up* (n=12) *
Overall / time-to-progression	17	50	50	83	166	199
Severe treatment toxicities	186	558	558	929	1858	2229

* Refers to number of prognostic factors included in the analysis

We estimated the sample size assuming an overall event rate for overall survival, time-to-progression and severe treatment toxicities of 0.60 and 0.86, and 0.14, a median follow-up time for overall survival and time-to-progression of 1.36 and 0.86 years, respectively [5]. Existing prognostic models for overall survival and time-to-progression had C statistics of 0.75 and 0.63, respectively [60]. Sakata *et al.*, (2019) reported a prognostic model for severe treatment toxicities with a C statistic of 0.70 [40]. We used the C statistics of these existing models to derive an anticipated adjusted Cox-Snell R^2 ($R^2_{CS_{adj}}$) of 0.484, 0.505 and 0.047 for overall survival, time-to-progression and severe treatment toxicities, respectively [50].

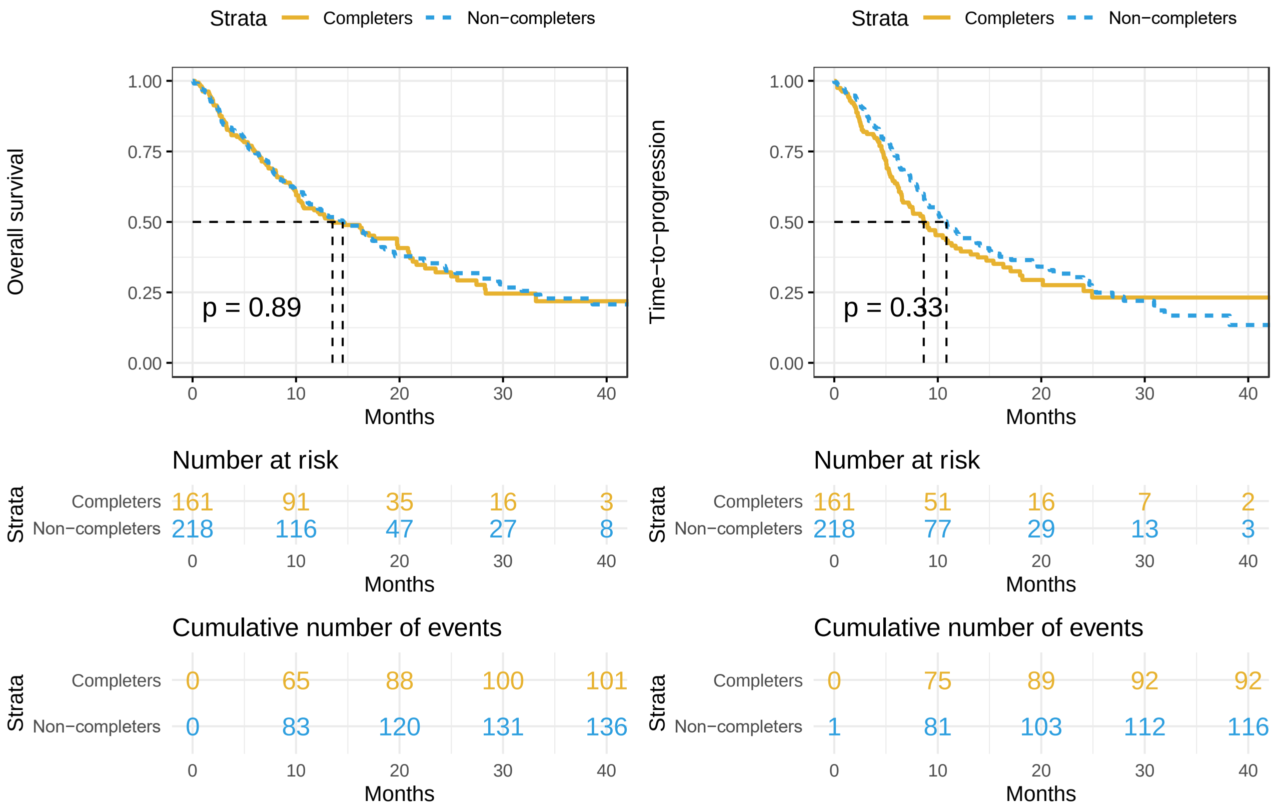

Appendix 5. Overall survival (left panel) and time-to-progression (right panel) in people with advanced NSCLC receiving immunotherapy compared between ePROM completers and non-completers.

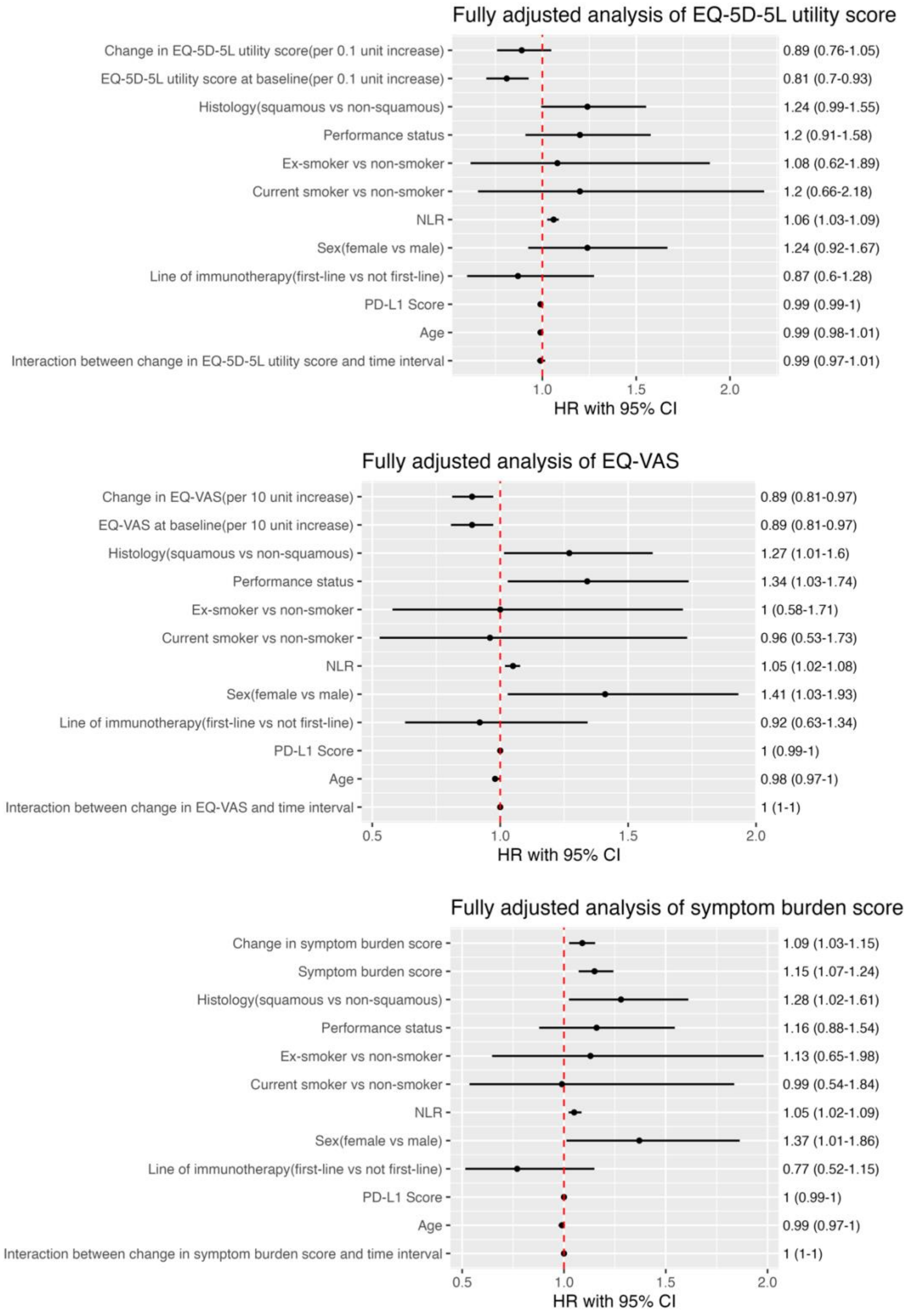

Appendix 6. Fully adjusted analysis of baseline ePROMs for predicting overall survival in people with NSCLC receiving immunotherapy. The ePROM prognostic factor is presented on the first row of each forest plot. Abbreviations: CI, confidence interval; ePROM, patient-reported outcome measure; HR, hazard ratio; NLR, neutrophil-to-lymphocyte ratio; PD-L1, programmed death-ligand 1.

Appendix 7. Prognostic value of ePROM scores for predicting overall survival in people with advanced NSCLC receiving immunotherapy. Similar results were observed regardless of different handling methods used to address missing data.

	N	HR (95% CI)§	P-value	N	HR (95% CI)§	P-value	N	HR (95% CI)§	P-value
	ePROM-only model*			Partially adjusted PROMs model†			Fully adjusted PROMs model‡		
ePROM at baseline									
EQ-5D utility score (per 0.1 unit increase)									
CCA	161	0.79 (0.69, 0.90)	<0.001	150	0.80 (0.70, 0.92)	0.0014	148	0.78 (0.68, 0.90)	<0.001
MI	379	0.84 (0.75, 0.94)	0.003	379	0.84 (0.75, 0.95)	0.006	379	0.84 (0.74, 0.95)	0.009
MIMI	379	0.84 (0.75, 0.94)	0.003	379	0.84 (0.75, 0.95)	0.006	379	0.84 (0.74, 0.95)	0.009
EQ-VAS (per 10 unit increase)									
CCA	161	0.93 (0.87, 1.00)	0.046	150	0.94 (0.88, 1.01)	0.10	148	0.92 (0.85, 1.00)	0.06
MI	379	0.93 (0.86,0.99)	0.04	379	0.93 (0.87,1.00)	0.07	379	0.92 (0.85, 1.00)	0.06
MIMI	379	0.93 (0.86, 0.99)	0.04	379	0.93 (0.87, 1.00)	0.07	379	0.92 (0.85, 1.00)	0.06
Symptom burden score									
CCA	161	1.12 (1.06, 1.21)	<0.001	150	1.11 (1.05, 1.19)	0.0011	148	1.13 (1.06, 1.21)	<0.001
MI	379	1.12 (1.05, 1.19)	0.0013	379	1.11 (1.04, 1.19)	0.003	379	1.13 (1.04, 1.22)	0.004
MIMI	379	1.12 (1.05, 1.20)	0.0012	379	1.11 (1.04, 1.19)	0.003	379	1.13 (1.04, 1.22)	0.004
Number of severe symptoms									
CCA	161	1.23 (1.09, 1.40)	0.002	150	1.20 (1.04, 1.37)	0.01	148	1.23 (1.07,1.43)	0.004
MI	379	1.23 (1.08, 1.41)	0.002	379	1.21 (1.05, 1.38)	0.007	379	1.23 (1.06, 1.42)	0.008
MIMI	379	1.23 (1.08, 1.41)	0.002	379	1.21 (1.05, 1.39)	0.007	379	1.23 (1.06, 1.42)	0.008
Change in ePROM									
EQ-5D utility score (per 0.1 unit increase)									
CCA	134	0.80 (0.71, 0.91)	< 0.001	125	0.85 (0.69, 1.05)	0.12	123	0.85 (0.68, 1.07)	0.16
MI	379	0.89 (0.77, 1.03)	0.11	379	0.90 (0.78, 1.04)	0.17	379	0.89 (0.76, 1.05)	0.17
MIMI	379	0.87 (0.77, 0.98)	0.11	379	0.88 (0.78, 0.99)	0.17	379	0.87 (0.76, 0.99)	0.17
EQ-VAS (per 10 unit increase)									
CCA	134	0.87 (0.80, 0.95)	0.002	125	0.91 (0.83, 0.99)	0.03	123	0.85 (0.77, 0.94)	0.002

MI	379	0.89 (0.83, 0.96)	0.005	379	0.90 (0.83, 0.97)	0.01	379	0.89 (0.81, 0.97)	0.01
MIMI	379	0.90 (0.83, 0.97)	0.01	379	0.90 (0.83, 0.98)	0.02	379	0.88 (0.79, 0.98)	0.03
Symptom burden score									
CCA	134	1.08 (1.02, 1.14)	0.006	125	1.07 (1.01, 1.13)	0.02	123	1.10 (1.02, 1.17)	0.008
MI	379	1.08 (1.03, 1.14)	0.003	379	1.08 (1.03, 1.14)	0.003	379	1.09 (1.03, 1.15)	0.008
MIMI	379	1.08 (1.03, 1.14)	0.004	379	1.08 (1.03, 1.14)	0.005	379	1.09 (1.02, 1.16)	0.01
Number of severe symptoms									
CCA	134	1.20 (1.05, 1.37)	0.009	125	1.16 (1.00, 1.34)	0.04	123	1.24 (1.04, 1.47)	0.02
MI	379	1.17 (1.03, 1.32)	0.02	379	1.16 (1.02, 1.32)	0.02	379	1.16 (1.01, 1.35)	0.04
MIMI	379	1.16 (1.02, 1.32)	0.03	379	1.16 (1.01, 1.32)	0.04	379	1.16 (1.00, 1.35)	0.06

Abbreviations: CCA, complete case analysis; CI, confidence interval; ePROM, electronic patient-reported outcome measure; HR, hazard ratio; MI, multiple imputation; MIMI, multiple imputation in conjunction with a missing indicator.

*For ePROM at baseline, ePROM-only models were univariable models. For change in ePROM, ePROM-only models were adjusted for baseline ePROM scores and an interaction term between change in ePROM score and months since baseline ePROM. All the analyses of MIMI additionally adjusted for a missing indicator of ePROM.

†Partially adjusted models were further adjusted for performance status and PD-L1 score.

‡Fully adjusted models were further adjusted for performance status, PD-L1 score, age, sex, smoking status, histology and line of immunotherapy.

§The HRs for MI and MIMI were the pool estimates of HRs derived from the imputed datasets.

The results of MI analyses are presented in Table 3 in the main text.

Appendix 8. Prognostic value of ePROMs for predicting time-to-progression in people with advanced NSCLC receiving immunotherapy. None of the ePROM scores was associated with time-to-progression.

	N	HR (95% CI)§	P-value	N	HR (95% CI)§	P-value	N	HR (95% CI)§	P-value
	ePROM-only model*			Partially adjusted PROMs model†			Fully adjusted PROMs model‡		
ePROM at baseline									
EQ-5D utility score (per 0.1 unit increase)									
CCA	161	1.03 (0.87, 1.22)	0.73	150	1.05 (0.87, 1.25)	0.62	148	1.04 (0.87, 1.25)	0.65
MI	379	1.04 (0.90, 1.20)	0.57	379	1.04 (0.90, 1.21)	0.58	379	1.06 (0.91, 1.23)	0.48
MIMI	379	1.04 (0.91, 1.20)	0.56	379	1.04 (0.90, 1.21)	0.57	379	1.06 (0.91, 1.23)	0.48
EQ-VAS (per 10 unit increase)									
CCA	161	0.98 (0.91, 1.06)	0.61	150	0.99 (0.91, 1.07)	0.76	148	0.98 (0.90, 1.07)	0.66
MI	379	0.98 (0.91, 1.05)	0.59	379	0.98 (0.91, 1.06)	0.68	379	0.98 (0.90, 1.06)	0.59
MIMI	379	0.98 (0.91, 1.06)	0.60	379	0.98 (0.91, 1.06)	0.69	379	0.98 (0.90, 1.06)	0.59
Symptom burden score									
CCA	161	1.00 (0.93, 1.08)	0.96	150	0.99 (0.92, 1.07)	0.88	148	0.99 (0.91, 1.08)	0.89
MI	379	0.99 (0.92, 1.06)	0.77	379	0.99 (0.92, 1.06)	0.77	379	0.98 (0.91, 1.06)	0.69
MIMI	379	0.99 (0.92, 1.06)	0.76	379	0.99 (0.92, 1.06)	0.76	379	0.98 (0.91, 1.06)	0.69
Number of severe symptoms									
CCA	161	0.96 (0.81, 1.15)	0.69	150	0.94 (0.79, 1.12)	0.50	148	0.94 (0.78, 1.14)	0.54
MI	379	0.94 (0.79, 1.11)	0.44	379	0.94 (0.79, 1.10)	0.43	379	0.92 (0.77, 1.10)	0.37
MIMI	379	0.99 (0.92, 1.06)	0.76	379	0.99 (0.92, 1.06)	0.76	379	0.92 (0.77, 1.10)	0.37
Change in ePROM									
EQ-5D utility score (per 0.1 unit increase)									
CCA	134	0.70 (0.60, 0.81)	< 0.001	125	0.69 (0.59, 0.80)	< 0.001	123	0.69 (0.59, 0.81)	< 0.001
MI	379	0.85 (0.75, 0.96)	0.01	379	0.85 (0.75, 0.96)	0.02	379	0.84 (0.73, 0.97)	0.02
MIMI	379	0.85 (0.75, 0.96)	0.01	379	0.85 (0.75, 0.96)	0.02	379	0.84 (0.73, 0.97)	0.02
EQ-VAS (per 10 unit increase)									
CCA	134	0.94 (0.85, 1.05)	0.27	125	1.00 (0.90, 1.12)	0.97	123	0.96 (0.85, 1.09)	0.53

MI	379	0.96 (0.89, 1.04)	0.32	379	0.98 (0.90, 1.07)	0.63	379	0.96 (0.88, 1.06)	0.43
MIMI	379	0.96 (0.89, 1.04)	0.31	379	0.98 (0.90, 1.07)	0.63	379	0.96 (0.88, 1.06)	0.44
Symptom burden score									
CCA	134	1.06 (0.99, 1.12)	0.07	125	1.04 (0.98, 1.11)	0.19	123	1.11 (1.02, 1.20)	0.01
MI	379	1.06 (0.999, 1.12)	0.053	379	1.05 (0.996, 1.11)	0.07	379	1.07 (1.00, 1.14)	0.04
MIMI	379	1.06 (0.9998, 1.12)	0.051	379	1.05 (0.996, 1.12)	0.07	379	1.07 (1.00, 1.14)	0.04
Number of severe symptoms									
CCA	134	1.15 (0.97, 1.35)	0.11	125	1.10 (0.92, 1.31)	0.35	123	1.28 (1.02, 1.60)	0.03
MI	379	1.10 (0.95, 1.27)	0.20	379	1.09 (0.94, 1.27)	0.24	379	1.11 (0.94, 1.31)	0.21
MIMI	379	1.10 (0.95, 1.27)	0.20	379	1.09 (0.94, 1.27)	0.25	379	1.11 (0.94, 1.31)	0.22

Abbreviations: CCA, complete case analysis; CI, confidence interval; ePROM, patient-reported outcome measure; HR, hazard ratio; MI, multiple imputation; MIMI, multiple imputation in conjunction with a missing indicator.

*For ePROM at baseline, ePROM-only models were univariable models in the complete cases analyses. For change in ePROM, ePROM-only models adjusted for baseline ePROM value, an interaction term between change in ePROM score and months since baseline ePROM completion at the time of first ePROM completion during follow-up. All the analyses of MIMI additionally adjusted for a missing indicator of ePROM.

†Partially adjusted PROMs models were further adjusted for performance status and PD-L1 score.

‡Fully adjusted PROMs models were further adjusted for performance status, PD-L1 score, age, sex, smoking status, histology and line of immunotherapy.

§The HRs for MI and MIMI were the pool estimates of HRs derived from the imputed datasets.

The results of MI analyses are presented in Table 3 in the main text.

Appendix 9. Prognostic value of ePROM scores for predicting severe treatment toxicities in advanced NSCLC receiving immunotherapy. None of the ePROM scores was associated with severe treatment toxicities.

	N	OR (95% CI)§	P-value	N	OR (95% CI)§	P-value	N	OR (95% CI)§	P-value
	ePROM-only model*			Partially adjusted model†			Fully adjusted model‡		
ePROM at baseline									
EQ-5D utility score (per 0.1 unit increase)									
CCA	161	1.34 (0.89, 2.03)	0.16	150	1.13 (0.75, 1.71)	0.55	148	1.09 (0.68, 1.76)	0.71
MI	379	1.02 (0.99, 1.04)	0.24	379	1.22 (0.84, 1.78)	0.31	379	1.20 (0.80, 1.81)	0.38
MIMI	379	1.02 (0.99, 1.04)	0.24	379	1.01 (0.99, 1.04)	0.3	379	1.01 (0.99, 1.04)	0.36
EQ-VAS (per 10 unit increase)									
CCA	161	0.95 (0.80, 1.14)	0.59	150	0.87 (0.72, 1.06)	0.18	148	0.86 (0.70, 1.07)	0.18
MI	379	0.99 (0.98, 1.01)	0.50	379	0.93 (0.79, 1.10)	0.39	379	0.93 (0.77, 1.11)	0.42
MIMI	379	0.99 (0.98, 1.01)	0.50	379	0.93 (0.79, 1.10)	0.39	379	0.93 (0.77, 1.11)	0.43
Symptom burden score									
CCA	161	0.87 (0.70, 1.07)	0.18	150	0.89 (0.70, 1.13)	0.31	148	0.90 (0.69, 1.18)	0.45
MI	379	0.92 (0.78, 1.08)	0.31	379	0.93 (0.78, 1.11)	0.43	379	0.91 (0.75, 1.11)	0.37
MIMI	379	0.92 (0.78, 1.08)	0.31	379	0.93 (0.78, 1.11)	0.43	379	0.91 (0.75, 1.12)	0.38
Number of severe symptoms									
CCA	161	0.60 (0.30, 1.19)	0.15	150	0.55 (0.23, 1.33)	0.19	148	0.60 (0.24, 1.49)	0.27
MI	379	0.71 (0.41, 1.23)	0.22	379	0.73 (0.41, 1.29)	0.28	379	0.69 (0.38, 1.29)	0.25
MIMI	379	0.71 (0.41, 1.23)	0.22	379	0.73 (0.41, 1.29)	0.28	379	0.70 (0.38, 1.30)	0.26
Change in ePROM									
EQ-5D utility score (per 0.1 unit increase)									
CCA	134	1.33 (0.80, 2.21)	0.26	125	1.33 (0.77, 2.28)	0.31	123	1.21 (0.67, 2.19)	0.52
MI	379	1.01 (0.98, 1.03)	0.64	379	1.01 (0.98, 1.03)	0.71	379	1.00 (0.98, 1.03)	0.78
MIMI	379	1.01 (0.98, 1.03)	0.64	379	1.01 (0.98, 1.03)	0.72	379	1.00 (0.98, 1.03)	0.78
EQ-VAS (per 10 unit increase)									
CCA	134	1.02 (0.81, 1.29)	0.84	125	1.03 (0.80, 1.32)	0.83	123	1.09 (0.81, 1.47)	0.56
MI	379	1.00 (0.98,1.02)	0.95	379	1.00 (0.98,1.02)	0.99	379	1.00 (0.98,1.02)	0.92

MIMI	379	1.00 (0.98,1.02)	0.96	379	1.00 (0.98, 1.02)	0.96	379	1.00 (0.98, 1.02)	0.94
Symptom burden score									
CCA	127	0.97 (0.81, 1.15)	0.71	125	0.99 (0.84, 1.17)	0.93	123	0.92 (0.75, 1.13)	0.4
MI	379	0.95 (0.82, 1.11)	0.52	379	0.95 (0.82, 1.11)	0.52	379	0.91 (0.76, 1.10)	0.34
MIMI	379	0.93 (0.77, 1.12)	0.45	379	0.93 (0.77, 1.12)	0.45	379	0.89 (0.72, 1.12)	0.31
Number of severe symptoms									
CCA	127	1.02 (0.69, 1.51)	0.92	125	1.10 (0.75, 1.62)	0.63	123	1.04 (0.66, 1.62)	0.87
MI	379	0.97 (0.67, 1.42)	0.89	379	0.98 (0.67, 1.43)	0.9	379	0.96 (0.62, 1.49)	0.85
MIMI	379	0.97 (0.67, 1.42)	0.88	379	0.98 (0.67, 1.42)	0.9	379	0.96 (0.62, 1.48)	0.84

Abbreviations: CCA, complete case analysis; CI, confidence interval; ePROMs, patient-reported outcome measures; MI, multiple imputation; MIMI, multiple imputation in conjunction with a missing indicator; OR, odds ratio.

*For ePROM at baseline, ePROM-only models were univariable models in the complete cases analyses. For change in ePROM, ePROM-only models adjusted for baseline ePROM value, an interaction term between change in ePROM score and months since baseline ePROM completion at the time of first ePROM completion during follow-up. All the analyses of MIMI additionally adjusted for a missing indicator of ePROM.

†Partially adjusted models were further adjusted for performance status and PD-L1 score.

‡Fully adjusted models were further adjusted for performance status, PD-L1 score, age, sex, smoking status, histology and line of immunotherapy.

§The ORs for MI and MIMI were the pool estimates of ORs derived from the imputed datasets.

The results of MI analyses are presented in Table 3 in the main text.

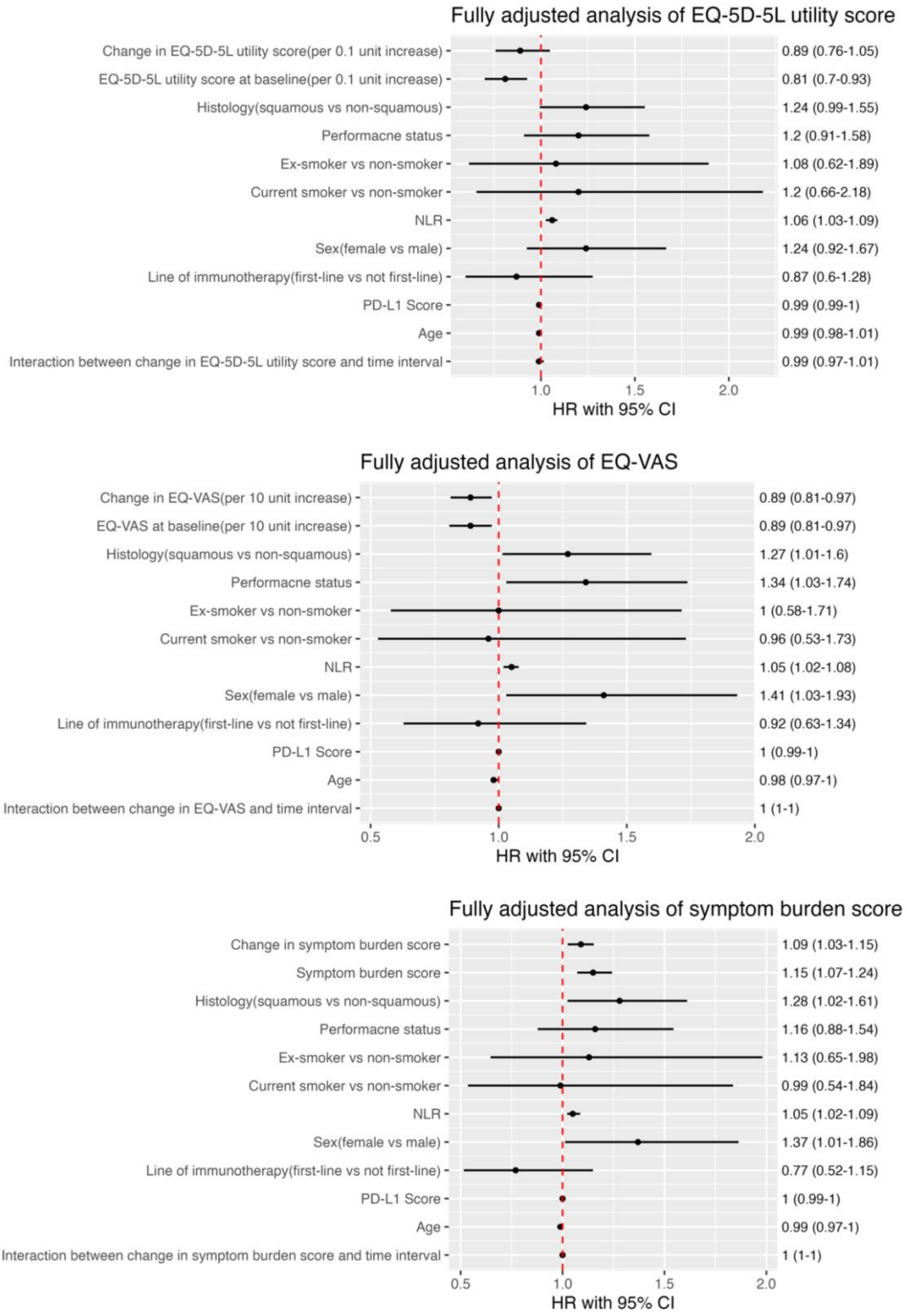

Appendix 10. Fully adjusted analysis of change in ePROMs for predicting overall survival in people with NSCLC receiving immunotherapy. The ePROM prognostic factor is presented on the first row of each forest plot. Abbreviations: CI, confidence interval; ePROM, patient-reported outcome measure; HR, hazard ratio; NLR, neutrophil-to-lymphocyte ratio ; PD-L1, programmed death-ligand 1.

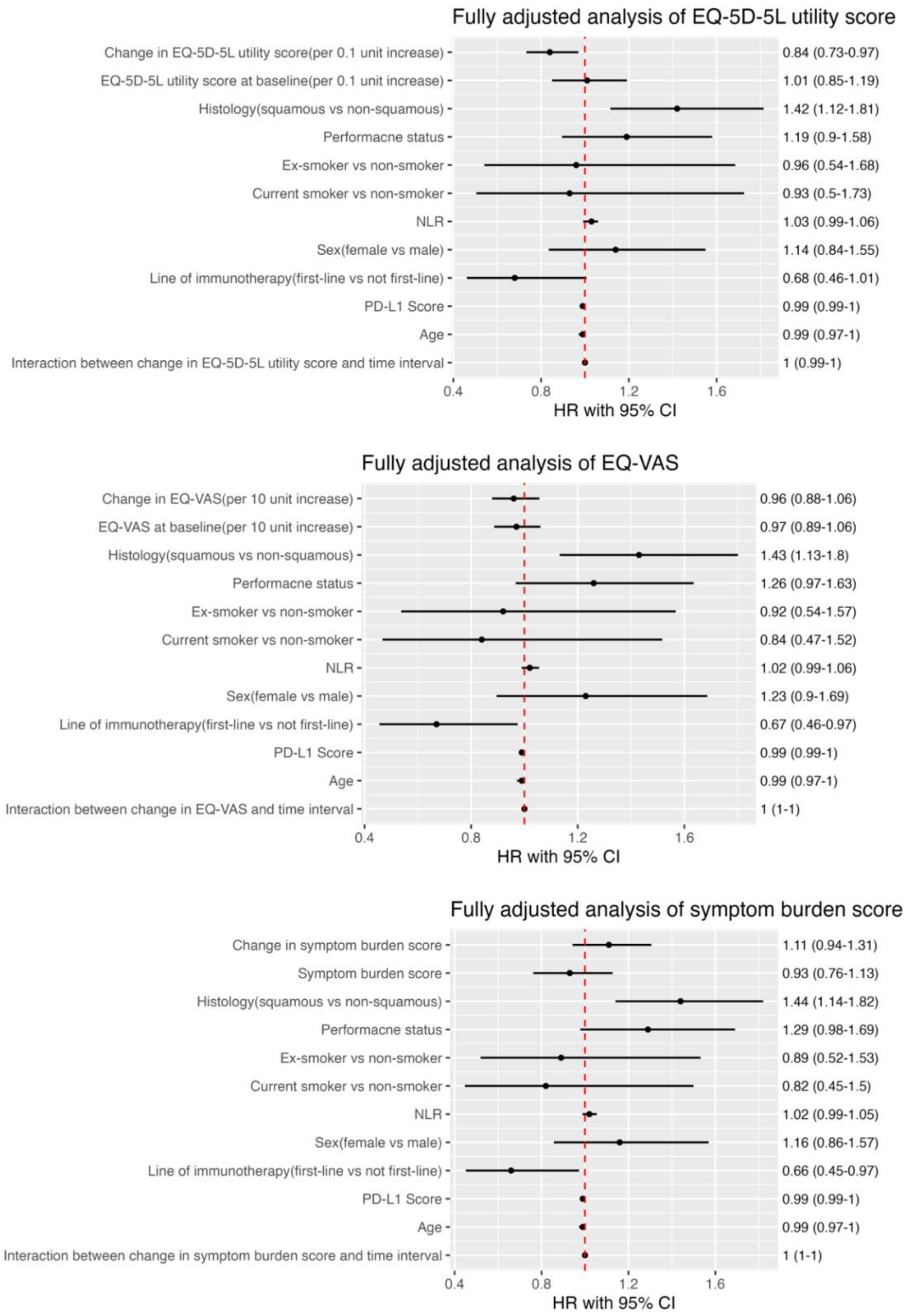

Appendix 11. Fully adjusted analysis of change in ePROMs for predicting time-to-progression in people with NSCLC receiving immunotherapy. The ePROM prognostic factor is presented on the first row of each forest plot. Abbreviations: CI, confidence interval; ePROM, patient-reported outcome measure; HR, hazard ratio; NLR, neutrophil-to-lymphocyte ratio; PD-L1, programmed death-ligand 1

Appendix 12. Prognostic value of ePROMs in people with advanced NSCLC receiving first line immunotherapy (n = 320). The results were obtained from the pooled estimates after multiple imputation.

Prognostic factor of interest	**Overall survival**		**Time-to-progression**		**Severe treatment toxicities**	
	HR (95%CI)	P-value	HR (95%CI)	P-value	OR (95%CI)	P-value
*ePROM at baseline**						
EQ-5D-5L utility score (per 0.1 unit increase)	0.85 (0.73, 1.00)	0.057	1.10 (0.90, 1.35)	0.34	1.22 (0.78, 1.90)	0.39
EQ-VAS (per 10 unit increase)	0.93 (0.85, 1.02)	0.13	0.98 (0.89, 1.07)	0.64	0.88 (0.71, 1.08)	0.15
Symptom burden score	1.10 (1.03, 1.18)	0.009	0.96 (0.88, 1.05)	0.4	0.90 (0.72, 1.13)	0.11
Number of severe symptoms	1.20 (1.03, 1.40)	0.02	0.92 (0.74, 1.15)	0.48	0.68 (0.34, 1.33)	0.26
Change in ePROM†						
EQ-5D-5L utility score (per 0.1 unit increase)	0.89 (0.74, 1.07)	0.22	0.83 (0.68, 1.00)	0.06	1.00 (0.97, 1.03)	0.87
EQ-VAS (per 10 unit increase)	0.84 (0.75, 0.94)	0.004	0.93 (0.82, 1.05)	0.22	1.00 (0.98, 1.03)	0.73
Symptom burden score	1.08 (1.02, 1.15)	0.018	1.08 (1.00, 1.16)	0.048	0.92 (0.76, 1.11)	0.39
Number of severe symptoms	1.15 (0.98, 1.35)	0.1	1.09 (0.90, 1.31)	0.38	1.01 (0.64, 1.60)	0.96

Abbreviations: CI, confidence interval; ePROMs, patient-reported outcome measures; EQ-5D-5L, EuroQoL five dimension five level; EQ-VAS, EuroQoL Visual Analogue Scale HR, hazard ratio; OR, odds ratio.

* The analysis of baseline ePROMs adjusted for performance status, PD-L1 score, age, sex, smoking status and histology.

† The analysis of change in ePROMs adjusted for performance status, PD-L1 score, age, sex, smoking status, histology, corresponding baseline ePROM score and an interaction term between change in ePROM score and months from baseline to the first completion of ePROMs during follow-up.

Appendix 13. The REMARK checklist

	Item to be reported	Page no.
**INTRODUCTION**		
1	State the marker examined, the study objectives, and any pre-specified hypotheses.	4
**MATERIALS AND METHODS**		
Patients		
2	Describe the characteristics (e.g., disease stage or co-morbidities) of the study patients, including their source and inclusion and exclusion criteria.	5
3	Describe treatments received and how chosen (e.g., randomized or rule-based).	5
Specimen characteristics		
4	Describe type of biological material used (including control samples) and methods of preservation and storage.	N/A
Assay methods		
5	Specify the assay method used and provide (or reference) a detailed protocol, including specific reagents or kits used, quality control procedures, reproducibility assessments, quantitation methods, and scoring and reporting protocols. Specify whether and how assays were performed blinded to the study endpoint.	6-7
Study design		
6	State the method of case selection, including whether prospective or retrospective and whether stratification or matching (e.g., by stage of disease or age) was used. Specify the time period from which cases were taken, the end of the follow-up period, and the median follow-up time.	5
7	Precisely define all clinical endpoints examined.	6
8	List all candidate variables initially examined or considered for inclusion in models.	6-7
9	Give rationale for sample size; if the study was designed to detect a specified effect size, give the target power and effect size.	9
Statistical analysis methods		
10	Specify all statistical methods, including details of any variable selection procedures and other model-building issues, how model assumptions were verified, and how missing data were handled.	8-9
11	Clarify how marker values were handled in the analyses; if relevant, describe methods used for cutpoint determination.	6-7
**RESULTS**		
Data		
12	Describe the flow of patients through the study, including the number of patients included in each stage of the analysis (a diagram may be helpful) and reasons for dropout. Specifically, both overall and for each subgroup extensively examined report the numbers of patients and the number of events.	N/A
13	Report distributions of basic demographic characteristics (at least age and sex), standard (disease-specific) prognostic variables, and tumor marker, including numbers of missing values.	9-11
Analysis and presentation		
14	Show the relation of the marker to standard prognostic variables.	13-14
15	Present univariable analyses showing the relation between the marker and outcome, with the estimated effect (e.g., hazard ratio and survival probability). Preferably provide similar analyses for all other variables being analyzed. For the effect of a tumor marker on a time-to-event outcome, a Kaplan-Meier plot is recommended.	Apps. 7, 8, 9
16	For key multivariable analyses, report estimated effects (e.g., hazard ratio) with confidence intervals for the marker and, at least for the final model, all other variables in the model.	14
17	Among reported results, provide estimated effects with confidence intervals from an analysis in which the marker and standard prognostic variables are included, regardless of their statistical significance.	Apps. 6, 10, 11
18	If done, report results of further investigations, such as checking assumptions, sensitivity analyses, and internal validation.	Apps. 7, 8, 9, 12
**DISCUSSION**		
19	Interpret the results in the context of the pre-specified hypotheses and other relevant studies; include a discussion of limitations of the study.	14-16
20	Discuss implications for future research and clinical value.	15-16
